# Comment on “A Cluster of Three Cases of* Hantavirus* Pulmonary Syndrome among Canadian Military Personnel”

**DOI:** 10.1155/2016/7458409

**Published:** 2016-12-20

**Authors:** Jan Clement, Piet Maes, Veroniek Saegeman, Katrien Lagrou, Marc Van Ranst, Åke Lundkvist

**Affiliations:** ^1^National Reference Centre for Hantavirus Infections, Laboratory of Clinical and Epidemiological Virology, Rega Institute for Medical Research, University Hospitals of Leuven, Leuven, Belgium; ^2^Laboratory Medicine and Department of Microbiology and Immunology, University Hospitals of Leuven, Leuven, Belgium; ^3^Zoonosis Science Center, Department of Medical Biochemistry and Microbiology, Uppsala University and Laboratory of Clinical Microbiology, Uppsala University Hospital, Uppsala, Sweden

We wish to congratulate Parkes et al. for their interesting findings on hantavirus pulmonary syndrome (HPS). They reported about three cases among Canadian military, who have fallen ill almost simultaneously about one month after participating in the same grand scale manoeuvres in Alberta, Canada, spring 2015 [1]. We want to extend their experience with similar findings, likewise in Canadian and in other military.

To our knowledge, the first (1988) clinically documented case of a hantavirus infection in a Canadian was also in the military, working however as a staff officer in the Supreme Headquarters of Allied Powers in Europe (SHAPE) in Belgium, and with no known prior history of rodent exposure [2]. However, he lived in a Belgian forested region highly endemic for Puumala virus (PUUV), and his IgM and IgG ELISA titers were highest for CG 13891, a PUUV strain, now rebaptized as CG Turnhout, and isolated already in 1985 from a bank vole, captured on a military exercise terrain near Turnhout, in North Belgium [3]. Moreover, this “SHAPE case” was later (1990) shown by neutralization testing (NT), the gold standard for confirming the infecting hantavirus species, to yield negative results for the prototype Korean hantavirus Hantaan virus (HTNV) and the worldwide rat-transmitted Seoul virus (SEOV) but to show titers of 80 for 80% neutralization and of 320 for 50% neutralization of PUUV (courtesy of James LeDuc, United States Army Medical Research Institute for Infectious Diseases (USAMRIID), unpublished 1990 NT results, sample Canada December 30, 1988, log # 682). Thus, this pioneer Canadian case was established to be in fact the first NT-confirmed PUUV infection in a New World patient.

Of even more interest, this same 1988 case appeared in retrospect to be one of the very first (and consequently then ill-understood) so-called “European HPS cases,” that is, the long-time underevaluated combination of acute lung injury (ALI) and acute kidney injury (AKI), with rapid deterioration of both lung and kidney function, necessitating often an Intensive Care Unit (ICU) admittance, mostly for acute lung problems. Indeed, he presented with rapidly worsening dyspnea, evanescent RX bilateral lung infiltrates suggestive for acute lung edema, and serious oxygen desaturation, prompting controlled oxygen and furosemide ICU therapy [2]. The concomitant distinct AKI needed however no specific renal replacement therapy (RRT), and both ALI and AKI resolved spontaneously and typically within two weeks [2]. Herewith, six years before the description of Sin Nombre virus- (SNV-) induced HPS as a newly recognized disease in Southwestern USA [4], a very similar entity of acute noncardiogenic lung edema, during a hantavirus infection in a previously healthy young adult, was already reported from Europe.

Another unusual hantavirus infection occurred also in a case in the Canadian military, working as a radio operator in the besieged city of Sarajevo during the Bosnian War [5]. In July 1992, he developed a severe AKI and worsening dyspnea, for which a helicopter evacuation to the Zagreb University Hospital was needed. Local Croatian physicians, although well familiarized with the European hantaviral AKI form, commonly called “hemorrhagic fever with renal syndrome” (HFRS), were puzzled by three consecutive IgG immunofluorescence assays (IFA), all negative for the then generally used hantavirus screening antigen, being the prototype Korean HTNV 76-118 strain. In this 1992 Croatian hospital report, pulmonary complications were not yet explicit, and finally no RRT was needed. Convalescent serum revealed in our hands high IgG IFA titers for two SEOV strains and for a then novel Dobrava virus (DOBV) strain, whereas IgM ELISA was clearly positive for HTNV 76-11. In PRNT (plaque-reduction neutralization test), the highest 50% neutralizing titer of 320 was for a SEOV strain (R22VP 30) [5]. Consequently, and particularly with regard to the patient's history of high exposure to wild rats, a rare diagnosis of European SEOV infection was put forward. However, the then recently isolated DOBV-Af strain could not be cultured in plaques for completing the battery of viruses for neutralization testing, leaving some lingering doubts about the exact origin of hantaviral infection. It became only clear after one of us (Åke Lundkvist, Sweden) had performed later a broader-spectrum focus-reduction neutralization test (FRNT) that this Canadian military had been infected in fact with a DOBV strain, explaining the more severe clinical course with dyspnea and thus constituting retrospectively this 1992 Sarajevo case as the first clinically documented and FRNT-confirmed DOBV case in Europe and the first “HPS” case caused by DOBV. However, a preliminary prior 1991 screening of mixed civilian and military European “HFRS” cases with an ELISA format, consisting of HTNV, SEOV, PUUV, and the then novel DOBV-Af antigen, had yielded already high titers to DOBV in 14/32 of Slovenian samples and in 2/27 of Belgian and Dutch samples [6].

Of interest, a very severe and rapid-onset ALI and AKI case was equally reported during the Bosnian War in a British soldier, prompting urgent air evacuation to his homeland, after emergency intubation and ventilation. A seriously deteriorating pulmonary, cardiac, and renal condition could be reversed only by a combination of high-tech ICU ventilation treatments in London, but RRT was not needed [7]. Here again, highest IgM and IgG ELISA titers were initially found against SEOV, but a later NT determined an underlying DOBV infection, confirming once again the severity potential of this hantavirus species (courtesy of Graham Lloyd, Public Health Laboratory, Porton Down, UK, unpublished data).

As noted by the current authors [1], a former “normal” HFRS scenario was demonstrated already in January 1990, when the first PUUV-induced HFRS outbreak in Germany, conspicuously enough exclusively in US military, was noted during NATO winter manoeuvres near Ulm, Baden-Württemberg (South Germany), totalling 15 IgM PUUV-confirmed and hospitalized AKI cases within 2 weeks [8]. The astonishing attack rate of 8.5% in the most stricken 177-member military logistic unit was in stark contrast with the fact that, during the entire same 1990 winter period, no civilian German HFRS (or HPS) case was registered in the same endemic Ulm region, demonstrating again that an unusual rodent exposure was and still is the most important risk factor for human hantavirus infections. Consequently, for one-month-long manoeuvres in the most endemic province of Canada, involving 6,750 military personnel from three different countries (Canada, USA, and UK), with supposedly the same degree of exposure, we wonder if the detection and description of a HPS cluster only in Canadian participants are to be ascribed solely to the clinical acumen of their compatriot physicians. Since moreover mild or asymptomatic SNV infections have been described, and even a rare SEOV-induced classic “HFRS” is not excluded in the New World (albeit much less frequently so in a forested biotope) [9], only extensive postmanoeuvre blood sampling could bring clarification. After the American 1990 PUUV outbreak in Germany, such an initiative yielded an additional 8 IgM-positive subclinical PUUV infections, whereas an ensuing US Army case-control study, the first of its kind in the young hantavirus era, singled out such now generally accepted risk factors as “sighting rodents” and “sleeping in hay” [8]. Of note, hantavirus screening for military operating in the European field should ideally comprise all hitherto known Old World hantaviral pathogens, being HTNV, SEOV, PUUV, DOBV, and even Tula virus (TULV), bearing in mind that all classic serological techniques can suffer from cross-reactions, including even PUUV cross-reacting with its American, but genetically related counterparts SNV and Andes virus (ANDV) [10].

With this brief recent historic overview, it should be clear that military operations in a European war- or manoeuvre-theatre enhance the risk for incurring not only HFRS, but also sometimes life-threatening “HPS” symptoms, most notably in a combination of both. This is an important reminder for military and even civilian, clinicians, and epidemiologists on both sides of the Atlantic. Although now a constant mantra in hantavirus literature since over 20 years [4], it remains a matter of debate of how two genetically related hantaviruses, after infection of humans via the same entry port (the lung) and inducing a pronounced but transient inflammatory “cytokine storm” with subsequent vascular leak, should consistently lead to two different clinical syndromes, or at least two syndromes with different, but often inappropriate, names, that is, HFRS and/or HPS, mainly because they occur on two different sides of the Atlantic [9].
